# Whole-genome analysis of *Streptococcus mutans* GDP01 isolate of dental caries tooth

**DOI:** 10.1128/mra.01057-24

**Published:** 2025-04-29

**Authors:** Guhanraj Radhamanalan, Suresh Mickymaray, Abdulaziz S. Alothaim, Noorah Alsowayeh, Dhanasekaran Dharumadurai

**Affiliations:** 1 Department of Microbiology, Bharathidasan University 30011, Tiruchirappalli, Tamil Nadu, India; 2 Department of Biology, College of Science, Al-Zulfi, Majmaah University 204569, Al Majmaah, Riyadh Province, Saudi Arabia; 3 National Repository for Microalgae and Cyanobacteria, Freshwater (NRMC-F) (Sponsored by the DBT, Govt. of India), Bharathidasan University 30011, Tiruchirappalli, Tamil Nadu, India; Rochester Institute of Technology, Rochester, New York, USA

**Keywords:** genomics, *S. mutans*, dental caries, virulance gene

## Abstract

Dental caries is a multifactorial infectious disease primarily driven by *Streptococcus mutans*. Here, we present the whole-genome sequence of *Streptococcus mutans* GDP01, which was isolated from the dental caries-infected children from Madurai, Tamil Nadu, India.

## ANNOUNCEMENT

**Fig 1:**
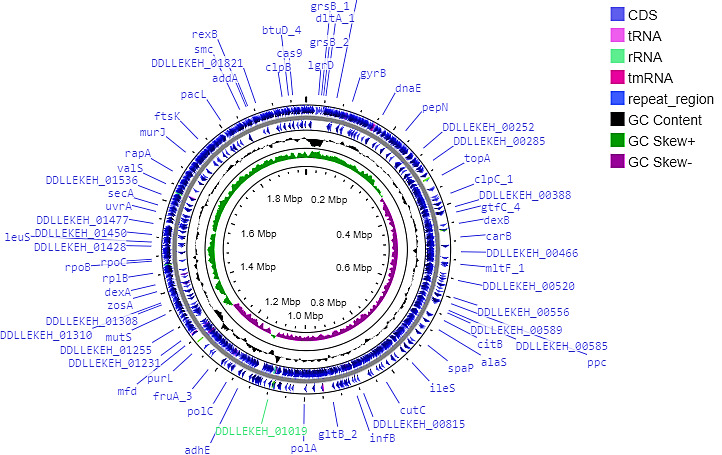
Circular genome map of *Streptococcus mutans* GDP01.

*Streptococcus mutans* is a major contributor to the development of dental cavities, which represent the most widespread chronic condition worldwide (1, 2). Dental caries is among the most prevalent infectious diseases in humans and frequently remains untreated, especially in less developed regions. *S. mutans*, a gram-positive, cocci-shaped bacterium, plays a pivotal role in the progression of dental caries (3). We collected dental caries samples aseptically by gently rubbing sterile cotton swabs over existing carious lesions and carefully rotating them across the tooth surface. Dental caries samples were obtained from children aged 10 to 19 years in Madurai, Tamil Nadu, India, with ethical approval from the Bharathidasan University (Ref. No. BDU/IEC/2020/3, dated 24 June 2020). The dental swab samples were serially diluted and inoculated onto Brain Heart Infusion (BHI) agar plates using the spread plate technique (4–6). The plates were incubated at 37°C for 24 hours, and the cultures were morphologically identified as *S. mutans*. Pure cultures of *S. mutans* GDP01 were inoculated into BHI broth, and genomic DNA (gDNA) was extracted using a bacterial gDNA extraction kit (Xploregen Discoveries). DNA quantification was carried out with a Qubit 3 fluorometer using a double-stranded DNA (dsDNA) high-sensitivity (HS) assay kit. A total of 100 µg of DNA was sheared into 200–300 bp fragments through the KAPA fragmentation process (7). The DNA fragments were purified using AMPure beads, and the sequencing library was prepared using the Qiagen NEBNext Ultra II DNA library preparation kit. The library was subsequently amplified using the NEBNext Ultra II Q5 Master Mix, following the manufacturer’s instructions. DNA was extracted in 15 µL of 0.1× Tris-EDTA buffer and measured using the dsDNA HS kit. For fragment analysis, the Agilent DNA 7500 chip was employed by Biokart India Pvt. Ltd., Bangalore. Sequencing was performed on an Illumina HiSeq 4000 system, generating 6.4 million paired-end reads (151 bp), 1,992, 406 bp with a 482.34× total sequencing yield. Quality control was conducted using FastQC v0.11.2 and MultiQC, while adapter removal and trimming were completed using TrimGalore v.0.6.4 (8). The raw reads were assembled using Unicycler v.0.4.8 (9). Genome completeness and contamination were assessed using QUAST v5.0.2 (10). It is used for assembly quality, confirming the genome length and contiguity. Species identification was confirmed through PubMLST (https://pubmlst.org/). Average nucleotide identity (ANI) analyses were performed (FastANI v.1.33) (11) to compare *S. mutans* GDP01 (https://www.ncbi.nlm.nih.gov/taxonomy/1309) with the reference strain, which showed 99.2% similarity to *S. mutans* UA159, confirming species identity. Unless otherwise stated, default parameters were used for all bioinformatics tools. *S. mutans* genome revealed a single contig with a genome size of 199 Mb and a GC content of 36.7% (Fig. 1). The Rapid Prokaryotic Genome Annotation Pipeline v1.14.6 predicted 1,883 coding sequences, 3 rRNA genes, 30 tRNA genes, and 1 tmRNA gene (N_50_:329,832 bp, L_50_:2). Virulence genes were identified using the Virulence Factor Database v.2024 and confirmed through BLASTp analysis (12, 13) ≥90%, sequence identity, and ≥80% query coverage. spaP (adhesion), luxS (biofilm formation), and ldh (acid production) genes were confirmed. Remarkably, we could not observe any genes that are nonpathogenic adaptation in *S. mutans* GDP01.

## Data Availability

This whole-genome project and associated data have been deposited in the NCBI database under the accession numbers Streptococcus mutans GDP01 Bio Project, PRJNA1162720; Bio Sample, SAMN43820816; and SRA, SRS22684413.
